# Preponderance of *CTLA4* Variation Associated With Autosomal Dominant Immune Dysregulation in the MYPPPY Motif

**DOI:** 10.3389/fimmu.2019.01544

**Published:** 2019-07-23

**Authors:** Owen M. Siggs, Amanda Russell, Davinder Singh-Grewal, Melanie Wong, Pearl Chan, Maria E. Craig, Ted O'Loughlin, Michael Stormon, Christopher C. Goodnow

**Affiliations:** ^1^Garvan Institute of Medical Research, Darlinghurst, NSW, Australia; ^2^Department of Ophthalmology, Flinders University, Adelaide, SA, Australia; ^3^The Children's Hospitals Network, The University of New South Wales, Sydney, NSW, Australia; ^4^St. Vincent's Clinical School, The University of New South Wales, Sydney, NSW, Australia

**Keywords:** CTLA4, ipilimumab, CTLA-4, *de novo* variant, autoimmune hepatitis, abatacept, juvenile rheumatoid arthritis (JRA)

## Abstract

One of the primary targets of immune checkpoint inhibition is the negative immune regulatory molecule CTLA-4. Immune-related adverse events are commonly observed following CTLA-4 inhibition in melanoma treatment, and a spectrum of these conditions are also observed in individuals with germline haploinsufficiency of *CTLA4*. Here we describe a heterozygous *de novo* missense variant of *CTLA4* in a young girl with childhood-onset autoimmune hepatitis and polyarthritis, the latter responding to treatment with CTLA-4-Ig fusion protein. This variant lay within the highly conserved MYPPPY motif of CTLA-4: a critical structural determinant of ligand binding, which is also bound by the anti-CTLA-4 monoclonal antibody ipilimumab. Within the spectrum of *CTLA4* variants reported, missense variants in the MYPPPY motif were overrepresented when compared to variants within a control population, highlighting the physiological importance of this motif in both the genetic and pharmacological regulation of autoimmunity and anti-tumor immunity.

## Introduction

Immune checkpoint inhibition, exemplified by inhibition of the CTLA-4 or PD-1 pathways, has become a pillar of cancer therapy. Monoclonal antibody-mediated inhibition of CTLA-4 has been shown to have striking anti-tumor effects, first in mice (1), and subsequently in humans (2). CTLA-4 itself is a negative regulator of T cell activation, with insufficiency of CTLA-4 associated with severe T cell-mediated autoimmunity in mice (3, 4), and with autosomal dominant immune dysregulation in humans (5, 6). The broad clinical spectrum of *CTLA4* insufficiency has close parallels with the immune-related adverse event profile observed in anti-CTLA-4 therapy (2), namely dermatologic and gastrointestinal (7), but also a significant proportion of carriers (>32%) with no clinical phenotype at all.

Despite the effectiveness of immune checkpoint inhibition in cancer therapy, acquired resistance can occasionally occur as a consequence of somatic mutations in the interferon receptor signaling or antigen presentation pathways (8). Similar processes may be at play in a recently described case of *CTLA4* haploinsufficiency associated with melanoma (9).

As larger numbers of *CTLA4* haploinsufficient cases are described, it has become possible to assess the distribution of variants across its distinct structural domains. Here we describe a case of *CTLA4* haploinsufficiency associated with a missense variant in the MYPPPY motif of CTLA-4, document their response to CTLA-4-Ig therapy, and present an analysis of the contribution of MYPPPY variants to the prevalence of *CTLA4*-associated immune dysregulation.

## Results

**Figure 1:**
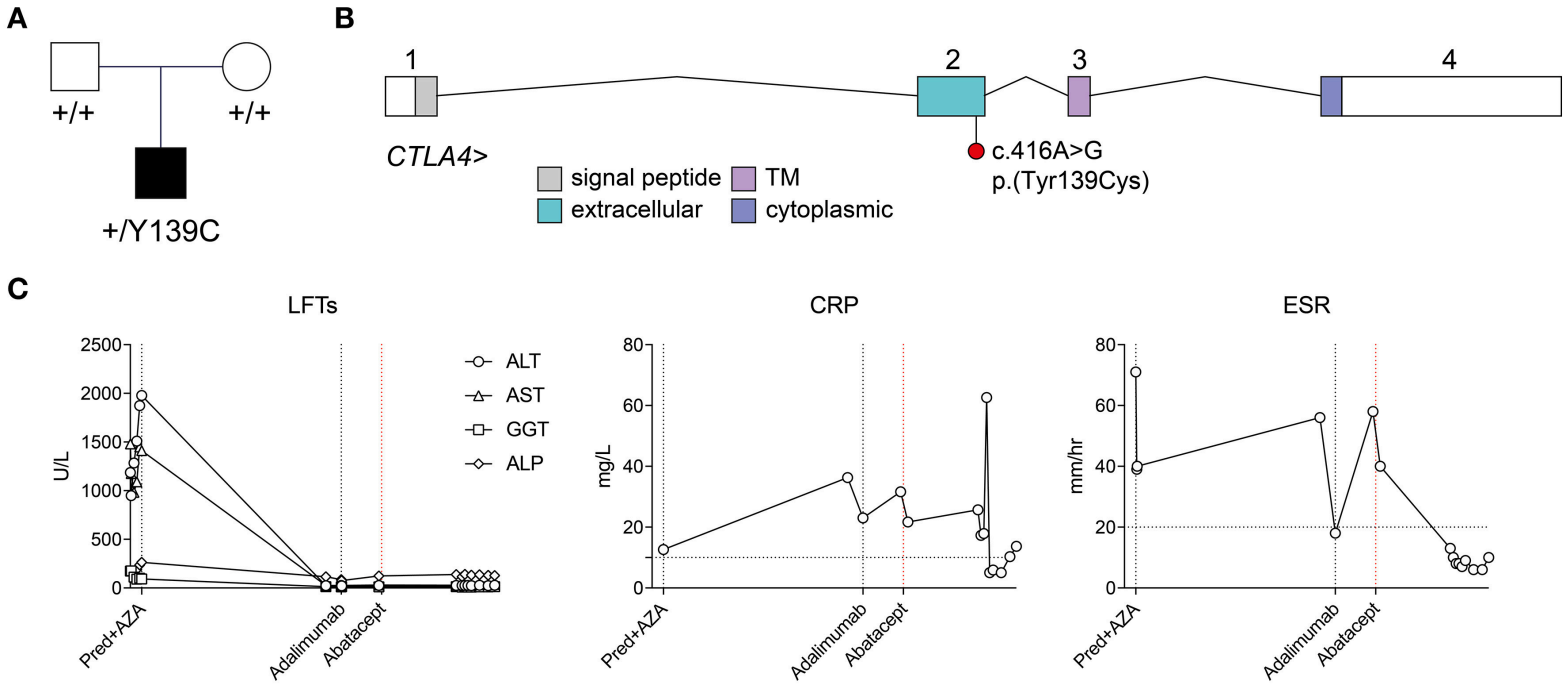
Identification of a *de novo* heterozygous p.(Tyr139Cys) variant in *CTLA4*, and response to abatacept treatment. **(A)** Pedigree and segregation of the *CTLA4* p.(Tyr139Cys) variant. **(B)** Exon structure of the *CTLA4* locus (indicating approximate boundaries of topological domains encoded by each exon) and location of the c.416A>G;p.Tyr139Cys) variant in exon 2. **(C)** Time course of liver enzymes, and inflammatory markers (CRP, ESR) and their response to systemic treatment. CRP, C-reactive protein; ESR, erythrocyte sedimentation rate; Pred+AZA, prednisolone and azathioprine. Horizontal broken lines indicate upper limits of normal.

As part of an effort to understand the genetic basis of exceptional immunological phenotypes, we ascertained a girl born to non-consanguineous parents with a constellation of immune-related conditions (Figure 1A). She was of European ancestry, with no family history of early-onset autoimmune or inflammatory disease. She was initially diagnosed with autoimmune hepatitis at 20 months of age, with serological studies revealing the presence of low-titer antinuclear autoantibodies (1:80 nucleolar), hypergammaglobulinemia, and IgA deficiency. Rheumatoid factor, anti-smooth muscle autoantibodies, and anti-liver-kidney-microsomal autoantibodies were not detected.

Following her initial diagnosis, the patient was treated with a tapering dose of oral prednisolone (from 20 mg per day), but due to steroid dependency azathioprine was introduced (20 mg per day). On this regime her liver enzymes normalized. However, 18 months later (aged 3 years, 2 months) she presented with polyarticular arthritis affecting large and small joints of the upper and lower limbs including the knees, ankles, elbows, and multiple small hand joints. This was initially treated with intra-articular steroid injections and subsequently a switch from azathioprine to mycophenolate with a good response. A flare of arthritis in a similar articular distribution occurred 6 months later (aged 3 years, 8 months), leading to repeated intra-articular injections and consideration of introduction of a biological agent to replace mycophenolate. Methotrexate was not used due to her underlying liver disease. As an alternative, subcutaneous adalimumab injections (20 mg every 2 weeks) were commenced 6 months later (aged 4 years, 4 months), and were partially effective, yet she continued to exhibit ongoing small joint disease of the metatarsophalangeal and metacarpophalangeal joints with stiffness and pain.

Genomic DNA samples isolated from peripheral blood from the patient and her parents were subjected to short-read genome sequencing. Variants were filtered by depth (≥ 10 reads), quality (QUAL ≥ 200), allele frequency (≤ 0.001 across 1000 Genomes, ExAC 0.2, and ESP populations), and medium or high impact (based on Ensembl classification categorization). When restricted to the canonical transcripts of 307 genes associated with inborn errors of immunity (10), with a Phred-scaled CADD score threshold of ≥ 15, only two variants were identified: the first a heterozygous missense variant in a gene associated with autosomal recessive kappa chain deficiency and also present in the unaffected father [*IGKC*, ENST00000390237.2:c.193C>A, ENSP00000374777.2:p. (Thr65Asn)], and the second a heterozygous variant in *CTLA4* [ENST00000302823.3:c.416A>G, ENSP00000303939.3:p.(Tyr139Cys)] (Figures 1B,C). This variant was absent from both biological parents, from the gnomAD r2.0.2 variant collection, and from a collection of 500 genomes sequenced contemporaneously at the same facility. The variant was predicted to be damaging by the PolyPhen-2 and SIFT algorithms, with a Phred-scaled CADD score of 24.1. Tyr139 itself lies within the highly conserved MYPPPY motif, and has previously been shown by site-directed alanine mutagenesis to be the most critical residue in this motif for binding of CTLA-4 and CD28 to their common ligands, CD80 and CD86 (11, 12). It has also recently been described in a kindred with incompletely penetrant splenomegaly, lymphadenopathy, hypogammaglobulinemia, and hypothyroidism (13).

Based on her genetic diagnosis, treatment of the patient's refractory juvenile rheumatoid arthritis (JRA) was switched from anti-TNF therapy (adalimumab) to CTLA-4-Ig (abatacept) therapy at age 4 years, 10 months. Initially started at 160 mg per month, her abatacept dosing was increased to 240 mg per month at age 5 years, 2 months. This regime initially gave a good response, yet was typically followed by breakthrough arthritis 2 weeks after each infusion. Since increasing her dosing regimen to 240 mg every 2 weeks (at age 5 years 4 months), she had sustained remission of her joint disease for more than 12 months.

In order to assess the relative contribution of variants to the prevalence of *CTLA4*-associated immune dysregulation, we assembled a collection of *CTLA4* variants from published reports (5–7, 13–15), from additional pathogenic and likely pathogenic variants deposited in ClinVar, and the *de novo CTLA4* variant described in this report.

**Figure 2:**
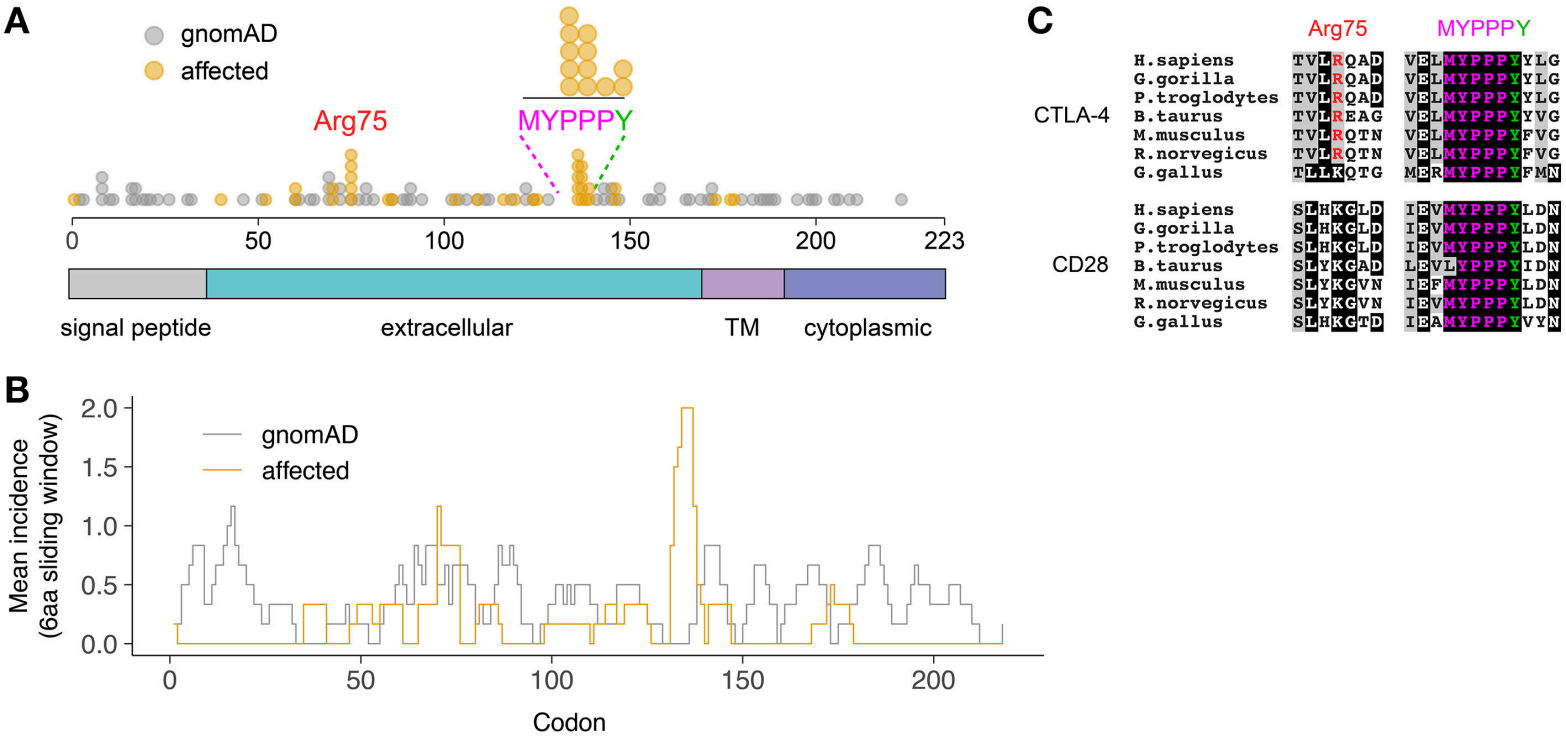
Preponderance of disease-associated variants in within the MYPPPY motif of CTLA-4. **(A)** Location of disease-associated *CTLA4* missense variants, demonstrating enrichment at Arg75, and the MYPPPY motif. Topological domains of the CTLA-4 protein are indicated in color below. **(B)** Mean incidence of disease-associated (affected) or control (gnomAD) missense variants across the *CTLA4* coding sequence using a 6-amino acid left-shifted sliding window. **(C)** Multi-species sequence conservation at Arg75 and the MYPPPY motif across CTLA-4 and its paralog, CD28.

**Figure 3:**
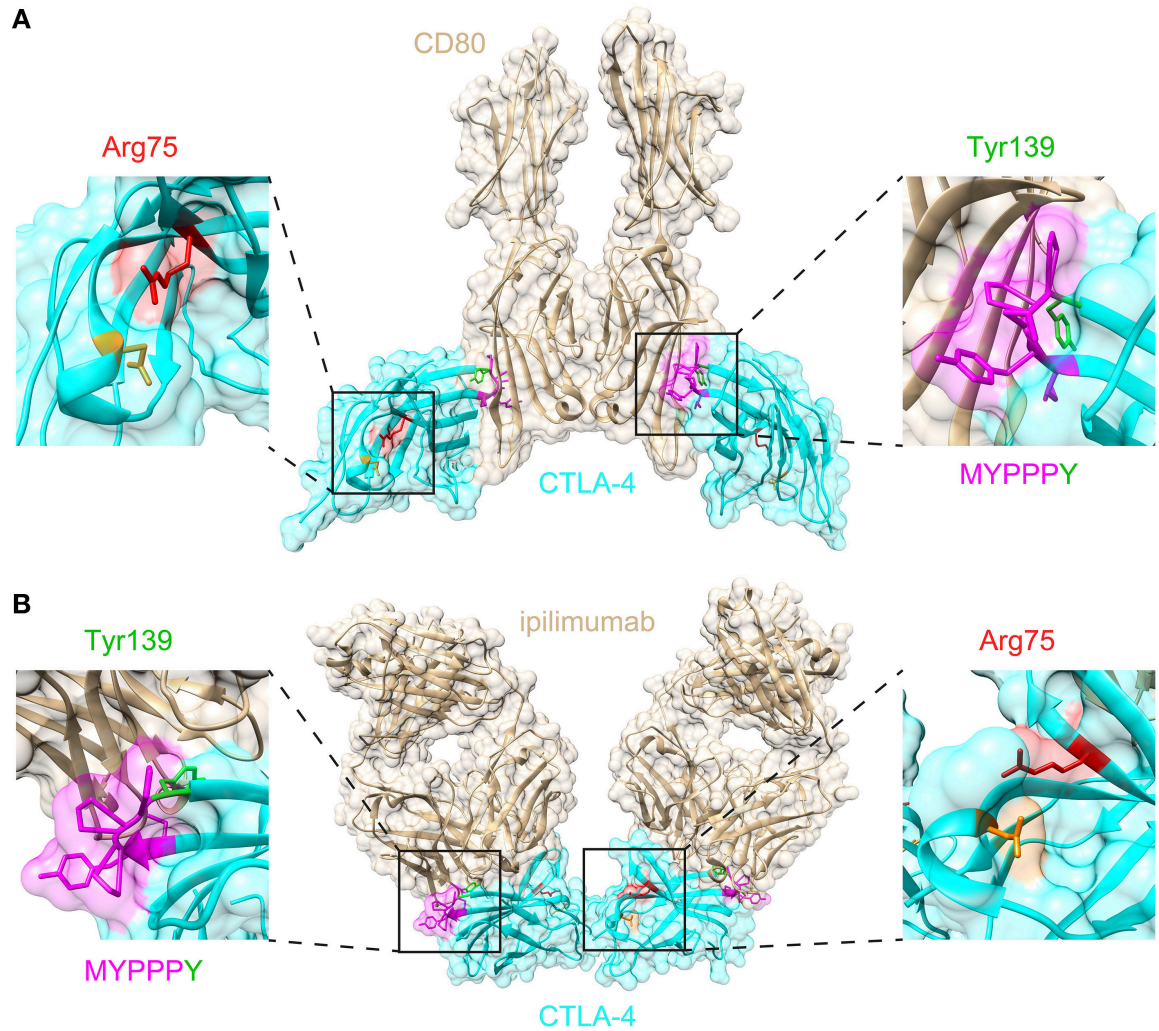
Structural significance of Arg75 and MYPPPY residues within CTLA-4. **(A)** Location of Arg75 and MYPPPY residues relative to the ligand-binding surface of CTLA-4 [PDB: 18IL (18)]. **(B)** Location of Arg75 and MYPPPY residues relative to the ipilimumab-binding surface of CTLA-4 [PDB: 5TRU (16)].

A total of 65 variants in unrelated families were identified (including the case described here), 26 of which (40%) were loss-of-function variants (defined as essential splice, frameshift, or stop gain variants), and 39 of which (60%) were missense variants (Figure 2A). Despite representing only 2.7% of the total protein sequence length (6/223 residues), variants in the MYPPPY motif represented 30.8% (12/39) of the disease-associated missense variant pool, including five independent missense variants at Pro136, four at Pro137, one at Pro138, and two at Tyr139 including the variant described here. A six-amino acid sliding window revealed a left-shifted peak incidence of disease-associated missense variants at codons 134–136 (Figure 2B). When compared with *CTLA4* missense variants within gnomAD controls (*n* = 82), missense variants in the MYPPPY motif were significantly more likely to be disease-associated than missense variants elsewhere in the *CTLA4* gene (Fisher's exact test, *P* = 3.342 × 10^−7^). The MYPPPY motif, including Tyr139, is highly conserved across both CTLA-4 and its immune stimulatory paralog CD28 (Figure 2C), and interfaces directly with both CD80 and with the CTLA-4 inhibitor ipilimumab (Figures 3A,B). A second enrichment of variants was noted at the Arg75 residue, where five independent missense variants were observed (5/39, 12.8%). Arg75 itself promotes the structural integrity of CTLA-4 by forming a critical salt bridge with Asp123 [Figures 3A,B; (16)].

## Discussion

We describe a single case of childhood-onset autoimmune hepatitis and polyarthritis associated with a *de novo CTLA4* variant. The *de novo CTLA4* variant described here occurs within the highly conserved MYPPPY motif, which contributes ~80% of the interfacial contacts with CD80 and CD86 ligands (17, 18). Binding of CTLA-4 to the anti-CTLA-4 monoclonal antibody ipilimumab results in the direct steric occlusion of this ligand binding surface, including the MYPPPY motif (16). The MYPPPY motif contributes ~60% of the total buried surface area provided by CTLA-4 in the CTLA-4:ipilimumab interaction, with Tyr139 being the dominant contributor within it (16). A second critical structural interface is the Arg75-Asp123 salt bridge, which when mutated results in loss of ipilimumab binding (16). A structural survey of missense variant distribution revealed that almost half of all disease-associated *CTLA4* missense variants (17/39, 43.6%) disrupted either the MYPPPY motif, or the Arg75-Asp123 salt bridge. This structural convergence emphasizes the importance of these two domains in both the genetic and pharmacological modulation of CTLA-4 function.

The same *CTLA4* variant as the one described here [c.416A>G;p.(Tyr139Cys)] has also been described in a second individual diagnosed with common variable immunodeficiency (13). This clinical presentation included splenomegaly, lymphadenopathy, hypogammaglobulinemia, and hypothyroidism, yet no evidence of hepatitis or polyarthritis. The *CTLA4* variant was inherited from the patient's unaffected father, with incomplete penetrance attributed to a maternally inherited *JAK3* variant present in the proband, identified through a search of 25 candidate genes. Although we could not exclude oligogenic inheritance in the case described here, the most parsimonious explanation is that a second rare variant was not required for disease. We did not perform any direct functional assessments, although previous experimental validation of the p.(Tyr139Cys) variant indicated reduced expression, an inability to bind CD80 or CD86, and absent suppressor activity in a T cell proliferation assay (13), consistent with earlier site-directed alanine mutagenesis experiments at the same codon (11, 12).

The p.(Tyr139Cys) variant exemplifies the poorly understood phenomena of incomplete penetrance and variable expressivity, which is commonly encountered in *CTLA4*-associated disease (7). Phenotypic manifestations of the same variant can range from hepatitis and polyarthritis in one individual, to common variable immunodeficiency in a second, or a complete absence of clinical signs and symptoms in a third. Liver involvement and arthritis are relatively rare clinical manifestations of *CTLA4*-associated immune dysregulation, respectively occurring in 12 and 14% of a series of 90 cases (7). *CTLA4* genotype does not appear to be important in this sense, with no observed association with disease phenotype, age of onset, or penetrance (7). We can only speculate as to the cause, although differences in genetic ancestry, either in the form of one or more common or rare genetic variants, may go some way toward explaining these differences, with environmental exposures sharing culpability. Beyond a single heterozygous variant of uncertain significance in *IGKC*, we did not identify any other rare predicted damaging variants in genes associated with Mendelian errors of immunity.

The present case also illustrates several important aspects of “precision medicine.” Genome sequencing provided a clear diagnosis for a previously idiopathic case, but more importantly revealed the pathogenesis: a single-gene defect leading to partial deficiency of an essential inhibitory immune receptor, CTLA-4. This result connected immediately to a strong laboratory and clinical evidence base that the patient would likely respond to targeted CTLA-4-Ig therapy: an agent that normally would not have been considered as first, second or third-line therapy in this case. In the patient's country of residence (Australia), the federal pharmaceutical benefits scheme normally requires JRA to be treated with methotrexate as first line, followed by anti-TNF or anti-IL-6R biological disease-modifying antirheumatic drugs, each requiring a 20-week therapeutic test period. Abatacept was not approved for JRA due to the low response rate in cases that had already responded poorly to anti-TNF therapy. Currently, there are no established mechanisms for government-subsidized treatment with abatacept in cases where a genetic diagnosis indicates it is the targeted treatment of choice.

## Materials and Methods

### Human Subjects

This study was carried out in accordance with the recommendations of the National Health and Medical Research Council's National Statement on Ethical Conduct in Human Research (M), with written informed consent from all subjects. All subjects gave written informed consent in accordance with the Declaration of Helsinki. The protocol was approved by the South Eastern Sydney Local Health District Human Research Ethics Committee.

### Genome Sequencing

Parent-proband trio genomes were sequenced on the Illumina HiSeq X platform using DNA isolated from whole blood (19). Libraries were generated using the Illumina TruSeq PCR-free protocol. Raw reads were aligned to the hs37d5 reference using BWA-MEM v0.7.10-r789, and sorted and duplicate-marked with Novosort v1.03.01 (Novocraft Technologies). The GATK suite v3.3-0-g37228af was used for local indel realignment and base quality score recalibration. gVCFs generated with GATK HaplotypeCaller were joint-called as trios using GATK GenotypeGVCFs, and variants recalibrated using GATK Variant Quality Score Recalibrator (VQSR). VCF files were annotated with Variant Effect Predictor (VEP) v76 using the LoFTEE and dbNSFP plugins (including CADD v1.3), and assembled into GEMINI databases (v0.18.3).

### *CTLA4* Variant Collection

*CTLA4* variants were collected from a series of published reports (5–7, 13–15), and from pathogenic and likely pathogenic variants listed on ClinVar (accessed October 3, 2018). Large deletion or duplication alleles were not included. Structural representations were generated from previously described structures [Protein Data Bank accession IDs: 18IL (18), and 5TRU (16)] using UCSF Chimera (20) and Adobe Illustrator.

## Data Availability

The variant described here has been deposited in ClinVar (accession number: SCV000891105).

## Ethics Statement

This study was carried out in accordance with the recommendations of the National Health and Medical Research Council's National Statement on Ethical Conduct in Human Research (2007), with written informed consent from all subjects. All subjects gave written informed consent in accordance with the Declaration of Helsinki. The protocol was approved by the South Eastern Sydney Local Health District Human Research Ethics Committee.

## Author Contributions

OS performed sequence and structure analysis, and wrote the paper with input from all authors. AR coordinated genome sequencing. DS-G, MW, PC, MC, TO'L, and MS coordinated patient recruitment and were responsible for clinical care. CG obtained funding and supervised research.

### Conflict of Interest Statement

The authors declare that the research was conducted in the absence of any commercial or financial relationships that could be construed as a potential conflict of interest.
